# Supplementary material for: Advanced Analysis and Validation of a microRNA Signature for Fanconi Anemia
Source: Genes (Basel). 2024 Jun 21;15(7):820. doi: 10.3390/genes15070820 (PMC11276059; doi:10.3390/genes15070820)
Supplement: Supplementary file 1 [file genes-15-00820-s001.zip › Table S1.pdf]

Table S1 - List of the 128 differentially expressed genes associated with the 17 pathways as in Tabble 2

Gene list

ACSL1
ACSL4
ADCY9
ALDH2
ANAPC7
ASH1L
ATP5E
B4GALT5
BAX
BIRC2
BIRC3
BIRC6
BTRC
BUB1B
CALM2
CAMK2D
CASP3
CCNB1
CCND1
CCND2
CCNE1
CCNE2
CCNG1
CCNG2
CDC25C
CDC27
CDC7
CDK1
CDK4
CDK6
CDKN1A
CDKN2A
CHEK1
COL4A1
COL6A2
COX6B1
CPEB2
CPEB3
CPEB4
CPT2
CUL2
CUL3
CUL5
DAG1
DNAH8
EGFR
ELOVL5
FADS2
FASN
FBXO5
FBXW7
FN1
GALNT1
GALNT2
GALNT3
GALNT7
GCNT3
GJA1
HERC2
HSD17B12
HSPG2
HTT
IGF1R
ITGA5
ITGA6
ITPR3
KAT2B
KMT2A
KMT2C
LAMA4
LAMB1
LAMB2
MAP3K1
MAPK1
MCM7
MDM2
NEDD4
NEDD4L
NFS1
NRAS
NSD1
PIK3CB
PKMYT1
PLOD2
PMAIP1
POLR2C
PPP1CB
PPP1CC
PPP2CA
PPP2R1B
PPP3CA
PPP3CB
PPP3R1
RPS6KA3
RRM2
RRM2B
SAE1
SERPINE1
SESN3
SETD2
SGOL1
SIAH1
SLK
SMC1A
SMC3
SOCS1
SOCS3
SUV39H2
TNFRSF10B
TP53
TP53I3
TRIP12
UBA3
UBE2D2
UBE2D3
UBE2E1
UBE2E3
UBE2K
UBE2R2
UBE2W
UBE3C
UQCRC1
WEE1
WHSC1L1
XIAP
YWHAG
YWHAQ
YWHAZ
