# Supplementary material for: Advanced Analysis and Validation of a microRNA Signature for Fanconi Anemia
Source: Genes (Basel). 2024 Jun 21;15(7):820. doi: 10.3390/genes15070820 (PMC11276059; doi:10.3390/genes15070820)
Supplement: Supplementary file 1 [file genes-15-00820-s001.zip › Table S2.pdf]

Table S2 - Core gene list shared by different pathways as from Table 2.

PATHWAYS NUMBER																
**1**	**2**	**3**	**4**	**5**	**6**	**7**	**8**	**9**	**10**	**11**	**12**	**13**	**14**	**15**	**16**	**17**
						BAX		BAX				BAX	BAX			BAX
					BIRC2											BIRC2
					BIRC3											BIRC3
						CASP3		CASP3	CASP3			CASP3				CASP3
						CCND1		CCND1	CCND1	CCND1			CCND1			CCND1
						CCND2		CCND2		CCND2						
			CCNE1			CCNE1		CCNE1		CCNE1						CCNE1
			CCNE2			CCNE2		CCNE2		CCNE2						CCNE2
			CDK1			CDK1		CDK1		CDK1					CDK1	
						CDK4		CDK4		CDK4			CDK4			CDK4
						CDK6		CDK6		CDK6			CDK6			CDK6
						CDKN1A		CDKN1A	CDKN1A	CDKN1A			CDKN1A			CDKN1A
						CDKN2A		CDKN2A		CDKN2A			CDKN2A			
						CHEK1		CHEK1		CHEK1						
									EGFR				EGFR		EGFR	
				FN1					FN1							FN1
	GALNT1															
	GALNT2															
	GALNT3															
	GALNT7															
			IGF1R						IGF1R				IGF1R			
				ITGA5					ITGA5							
				ITGA6												ITGA6
				LAMA4												LAMA4
				LAMB1												LAMB1
				LAMB2												LAMB2
			MAPK1					MAPK1	MAPK1				MAPK1		MAPK1	
										MCM7						
					MDM2	MDM2		MDM2	MDM2	MDM2			MDM2			
								NRAS	NRAS				NRAS		NRAS	
								PIK3CB	PIK3CB				PIK3CB			PIK3CB
			PPP1CB						PPP1CB							
			PPP1CC						PPP1CC							
						TP53		TP53	TP53	TP53		TP53	TP53			TP53
			YWHAG					YWHAG		YWHAG						
			YWHAQ					YWHAQ		YWHAQ						
			YWHAZ					YWHAZ		YWHAZ						
